# Effects of eye-acupuncture combined with rehabilitation training for poststroke dyskinesia: A protocol for systematic review and meta-analysis

**DOI:** 10.1097/MD.0000000000025036

**Published:** 2021-03-12

**Authors:** Qi Wang, Nanting Ma, Pengqin Wang, Mei Wang, Yan Shao, Xitong Zhao

**Affiliations:** a Liaoning University of Traditional Chinese Medicine; b Affiliated Hospital of Liaoning University of Traditional Chinese Medicine, Shenyang, China.

**Keywords:** dyskinesia, eye-acupuncture, meta-analysis, rehabilitation training, stroke, systematic review

## Abstract

**Introduction:**

Poststroke dyskinesia is the most common clinical symptom after stroke, which greatly affects the patients’ daily life. Eye-acupuncture is an effective method for stroke. And the rehabilitation training has been widely used for patients suffer from stroke. However, whether eye-acupuncture combined with rehabilitation training has greater clinical efficacy for poststroke dyskinesia is still unknown. Our aim in this systematic review was to evaluate the clinical efficacy of eye-acupuncture combined with rehabilitation training (EACRT) as a treatment for dyskinesia after stroke.

**Methods and analysis:**

We will search the following 4 databases of registered trials and 7 electronic databases from inception to March 2021:Cochrane Stroke Group, Cochrane Central Register of Controlled, the World Health Organization International Clinical Trials Registry Platform, the Chinese Clinical Trial Registry; PubMed, MEDLINE, Embase, CNKI, VIP, WanFang, and CBM. All relevant randomized controlled trials focus on EACRT will be included. The primary outcome will be the Fugl-Meyer Assessment. The Secondary outcomes will include Activity of Daily Living, clinical effective rate and the Visual Analogue Score. Two reviewers will independently conduct the Study selection and data extraction. The data synthesis and assessment of risk of bias will be performed by RevMan5.2.

**Ethics and dissemination:**

The ethical approval is unnecessary that systematic review is based on published articles other than patients. The results of this meta-analysis will be published in an open access (OA) journal according to the Preferred Reporting Item for Systematic Review and Meta-analysis (PRISMA).

**PROSPERO registration number:**

CRD42020168278.

## 1 Introduction

Stroke is a destructive disease features high morbidity and mortality and high recurrence rate with the main symptoms of sudden collapse, unconsciousness, facial palsy and hemiplegia, which has become the leading cause of death for elderly people. In China, more than 2.5 million people suffer from stroke every year.^[1]^ Poststroke dyskinesia is the most common clinical symptom, which greatly affects the patients’ daily life. Therefore, it is helpful that doctors apply effective strategies to improve the motor functions. Evidence-based medical studies show that early rehabilitation training for stroke patients is the most effective way to reduce the disability rate of patients.^[2]^ Timely improvement of stroke patients’ motor function is the key to improve patients’ activities of daily life and help them return to the society as soon as possible.

**Figure 1:**
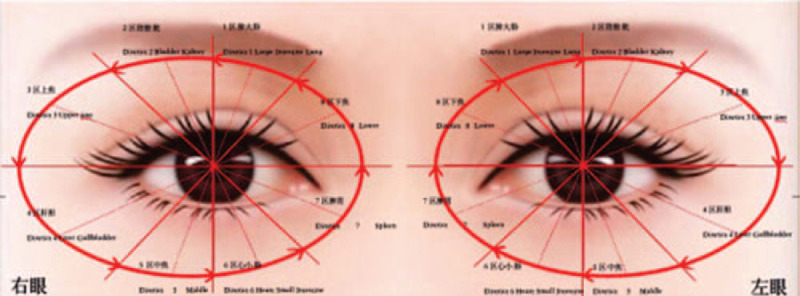
Diagram of acupoint location of eye-acupuncture.

As one of the main physical therapies in Traditional Chinese Medicine, acupuncture has been widely applied to improve motor function for more than hundreds of years in China.^[3]^ Eye-acupuncture originated by Peng Jingshan, a famous acupuncturist in China whose academic views were based on the theory of traditional Chinese medicine (TCM). According to the principle of picking points in 8 areas and thirteen points (specifically shown in Fig. 1) under TCM syndrome differentiation and diagnosis, acupuncture was performed in the area around the orbit, so as to improve the patient's clinical symptoms.^[4]^ Eye-acupuncture has been used in clinic for more than 40 years with advantages of convenient operation, economical cost, less adverse reactions and great clinical efficacy which is gradually recognize internationally.^[5]^ Therefore, eye-acupuncture combined with rehabilitation training (EART) can serve as an effective alternative to these conventional treatments.

The aim of this systematic review and meta-analysis was to confirm the effectiveness of EART in improving the motor functions for patients suffer from stroke and to provide basis for the clinical standard application of eye acupuncture.

## 2 Methods and analysis

### 2.1 Study registration

This protocol of meta-analysis has been registered in PROSPERO^[6]^ with registration number as CRD42020168278. The results of this systematic review will be published according to the Preferred Reporting Item for Systematic Review and Meta-analysis (PRISMA) statement guidelines.

### 2.2 Inclusion criteria

#### 2.2.1 Type of studies

The included articles will be all randomized controlled trials (RCT) focus on eye-acupuncture combined with rehabilitation training for dyskinesia after stroke. And there will be no restriction on language or publication status.

#### 2.2.2 Type of participants

Poststroke patients with dyskinesia between 30 to 80 years old will be included. No restriction on nation, gender or race.

#### 2.2.3 Type of interventions

The eye-acupuncture combined with rehabilitation training should be the major intervention in experimental group for the included articles and no restriction on the specific acupuncture points.

#### 2.2.4 Type of comparators

The interventions used in control group will be conventional rehabilitation, traditional Chinese medicine, western medicine, sham acupuncture or other common treatments.

#### 2.2.5 Types of outcome measurements

The primary outcome will be the Fugl-Meyer Assessment. The Secondary outcomes will include Activity of Daily Living, clinical effective rate and the Visual Analogue Score. Moreover, the adverse events will also be evaluated.

### 2.3 Exclusion criteria

The exclusion criteria include:

a) The dyskinesia is not caused by stroke but other reasons.
b) The trails are not randomized controlled trials but observational articles, reviews or other literature researches.
c) The intervention of experimental groups is only eye-acupunture or rehabilitation training without combined both of them.
d) The publications are duplicated in different databases or the clinical trails are not finished with no data could be extracted.
e) The full articles could not be acquired from OA articles website or email.

### 2.4 Search strategy

We will adopt the search strategy drafted by Cochrane Stroke Group. Articles that comprised randomized controlled trails of EART as treatment for improving the motor functions for patients suffer from stroke, will be collected in accordance with the following strategy:

a) We will search the Cochrane Stroke Group trials register for any trials with eye-acupuncture in the title or keyword sections.
b) We will search the following databases of registered trials: Cochrane Central Register of Controlled in the Cochrane Library, the World Health Organization International Clinical Trials Registry Platform, the Chinese Clinical Trial Registry (www.chictr.org.cn/searchproj.aspx).
c) We will also search the following electronic database: PubMed, MEDLINE, EMBASE, the China National Knowledge Infrastructure Database, the Chinese Science Journal Evaluation Reports database, WanFang Data and the China Biology Medicine.

### 2.5. Studies selection

2.5

**Figure 2:**
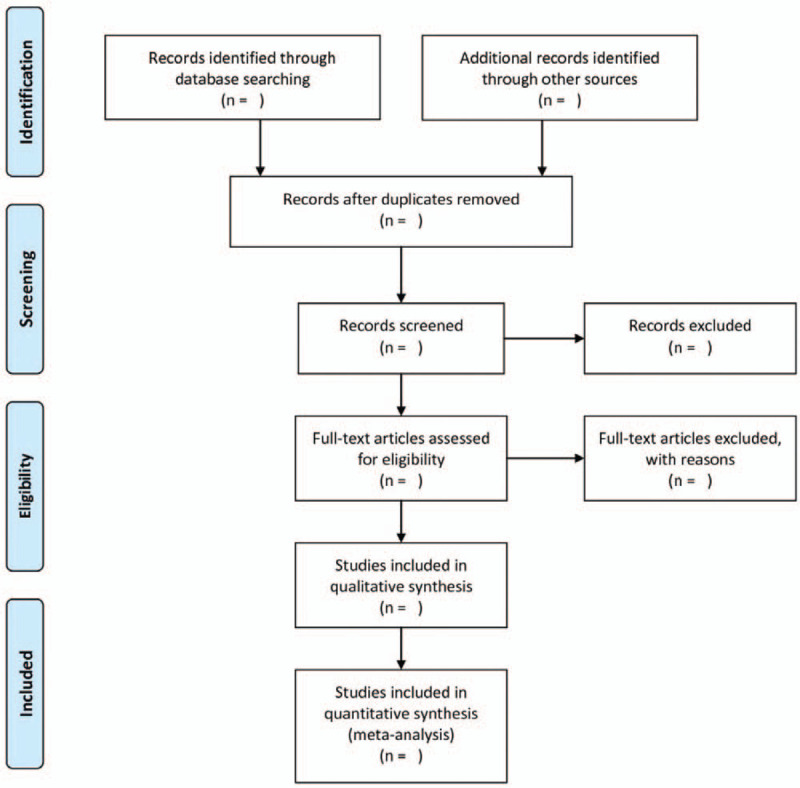
The flow chart.

Two review authors (QW, NTM) will search the electronic databases independently and included eligible articles according with the inclusion criteria. We will resolve the disagreements by discussion or consultation with the third author (PQW) if necessary. All the retrieved articles will be input in NoteExpress 3.2.0 for filtering the duplicated. The process of study selection will be implemented according to the PRISMA flow diagram (specifically shown in Fig. 2)

### 2.6 Data extraction

We will formulate a data extraction form that contains:

a) Characteristics of each article, including the title, and published year.
b) Characteristics of participants, including the sample size, and the average age.
c) The study design, interventions, and outcomes.

Two review authors (QW, NTM) will independently evaluate the study design, the criteria for participants, and the outcome measures. And they will extract the data and request the study's author for additional data. As for difference, the third author (PQW) will be consulted for eliminating disagreements.

### 2.7 Assessment of risk of bias

According to the Cochrane Handbook for Systematic Reviews of Interventions,^[7]^ we will assess the risk of bias for the included studies by the following methodological factors and graded each factors as “low” “high” or “unclear”:

a) Method of randomization.
b) Allocation concealment.
c) Blinding for participants and outcome assessors.
d) Intentional analysis.
e) Incomplete outcome data.
f) Selective outcome reporting.
g) Other bias.

We will formulate tables and 2 review authors (QW, NTM) will independently assess the risk of bias for each included articles. We will resolve the disagreements by discussion or consultation with the third author (PQW) if necessary.

### 2.8 Data analysis

We will use the comprehensive statistical software (RevMan5.2) to perform all the meta-analysis. Dichotomous outcome variables will be compared and its results will be presented as the odds ratio (OR). Continuous outcome variables will be analyzed by the standardized mean difference (SMD). We will use *I*^2^ statistics to evaluate the homogeneity. If the *I*^2^ < 50%, we will consider the outcome is homogeneous so the fixed-effects model would be used. And if the *I*^2^ > 50%, we could consider the outcome is heterogeneous so the random-effects model should be chosen. The pooled differences and 2 sides *P* values will be calculated for the level in statistical significance. When the *P* value <.05, the outcomes can be considered statistically significant. Meanwhile, we will also conduct the sensitivity analysis based on the cross validation. The narrative analysis will also be conducted if the results could not be mathematic analysis.

#### 2.8.1 Dealing with missing data

We will contact the studies’ authors for additional information by email if the included studies are short of essential data or the exist data is unclear. Moreover we will only analyze the available data if the authors does not reply and the potential impact for the missing data will be clear in discussion.

#### 2.8.2 Subgroup analysis

In order to investigate heterogeneity caused by the difference of studies, we will carry out the subgroup analysis in terms of intervention of control group (rehabilitation training, western medicine, traditional Chinese medicine, normal acupuncture, etc.), effects in ischemic and hemorrhagic stroke, effects of time of the start of acupuncture and effects of stroke severity.

#### 2.8.3 Sensitivity analysis

We will conduct the sensitivity analysis to examine the reliability of the study results. The studies features high bias such as open control, unclear of allocation concealment and no blinded outcome evaluation will be excluded separately for sensitivity analysis.

#### 2.8.4 Publication bias

We will perform a funnel plot to analyse publication bias for the included trials if the number of included articles is over 10.

### 2.9 Grading of recommendations assessment, development and evaluation

We will adopt the GRADE (Grading of Recommendations Assessment, Development, and Evaluation)^[8]^ to assess the outcomes in terms of publication bias, inconsistency, indirectness, imprecision and limitations as well as to rate the quality of outcomes as “high” or “moderate” or “low” or “very low”. We will show the assessment results in the attached file.

### 2.10 Ethics and dissemination

The ethical approval is unnecessary on account that systematic review is based on published articles other than patients. The results of this meta-analysis will be published in an open access (OA) journal according to the PRISMA.

## 3 Discussion

Eye-acupuncture therapy is kind of micro-needle therapy originally developed by the famous acupuncturist Peng Jingshan in China. His academic views inspired by the medical saint Hua Tuo's idea that “seeing the eyes can detect the internal diseases”.^[9]^ During the operation, acupuncture is performed in the periorbital acupoints after diagnosis of TCM syndrome differentiation, so as to improve the clinical symptoms of patients.^[10]^ Compared with conventional therapy, eye-acupuncture therapy has been used in clinic for more than 40 years with the advantages of convenient operation, economic cost and less adverse reactions. It has great effects in improving motor dysfunction after stroke and is gradually recognized by the international community. In recent years, there have been a large number of randomized controlled trials on the combination of eye acupuncture with rehabilitation training. We will conduct this meta-analysis to evaluate its efficacy and provide a basis for the clinical standard application of eye-acupuncture.

## 4 Strengths and limitations

The protocol of this systematic review has been registered in PROSPERO with registration number as CRD42020168278. Our meta-analysis will assess the efficacy and safety of eye-acupuncture combined with rehabilitation training (EACRT) for poststroke dyskinesia according to the PRISMA standards. And there exist the limitations for the included articles will only be written in English and Chinese, so there will be bias for the article selection.

## Author contributions

**Conceptualization:** Qi Wang, Pengqin Wang, Yan Shao.

**Data curation:** Qi Wang, Nanting Ma, Mei Wang, Xitong Zhao.

**Formal analysis:** Qi Wang, Mei Wang.

**Funding acquisition:** Pengqin Wang, Yan Shao, Xitong Zhao.

**Investigation:** Nanting Ma.

**Methodology:** Qi Wang, Nanting Ma, Mei Wang.

**Project administration:** Pengqin Wang, Yan Shao.

**Resources:** Qi Wang, Pengqin Wang, Yan Shao, Xitong Zhao.

**Software:** Qi Wang, Mei Wang.

**Supervision:** Pengqin Wang, Yan Shao.

**Validation:** Nanting Ma, Mei Wang, Xitong Zhao.

**Visualization:** Qi Wang, Nanting Ma, Mei Wang, Xitong Zhao.

**Writing – original draft:** Qi Wang, Nanting Ma.

**Writing – review & editing:** Qi Wang, Nanting Ma, Xitong Zhao.
